# Anemia in the Elderly

**DOI:** 10.1097/HS9.0000000000000040

**Published:** 2018-04-17

**Authors:** Domenico Girelli, Giacomo Marchi, Clara Camaschella

**Affiliations:** 1 Department of Medicine, Section of Internal Medicine, University of Verona, Verona, Italy; 2 Division of Genetics and Cell Biology, San Raffaele Scientific Institute, Milan, Italy.

## Abstract

Anemia affects a substantial fraction of the elderly population, representing a public health problem that is predicted to further increase in coming years because of the demographic drive. Being typically mild, it is falsely perceived as a minor problem, particularly in the elderly with multimorbidity, so that it often remains unrecognized and untreated. Indeed, mounting evidence indicates that anemia in the elderly (AE) is independently associated with disability and other major negative outcomes, including mortality. AE is generally multifactorial, but initial studies suggested that etiology remains unexplained in near one-third of cases. This proportion is consistently declining due to recent advances highlighting the role of several conditions including clonal hematopoiesis, “inflammaging,” correctable androgen deficiency in men, and under-recognized iron deficiency. Starting from a real-world case vignette illustrating a paradigmatic example of anemia in an elderly patient with multimorbidity, we review the main clinical and pathophysiological aspect of AE, giving some practical insights into how to manage similar cases.

## Case vignette

An 81-year-old man, nursing home resident, is known to be affected by multiple comorbidities including: postischemic chronic heart failure (CHF) with previous myocardial infarction 6 years earlier, reduced ejection fraction at echocardiography (40%), and 1 hospital admission 4 months earlier because of acute heart failure precipitated by a flu-like episode; type 2 diabetes mellitus; stable stage 2 chronic kidney disease (CKD) with estimated glomerular filtration rate of 60 mL/min; and bilateral coxarthrosis with reduced mobility. No substantial cognitive decline is evident. His chronic medications include aspirin 100 mg/day, lansoprazole 30 mg/day, bisoprolol 2.5 mg/day, ramipril 5 mg/day, furosemide 50 mg/day, and metformin 1000 mg/day. His periodical routine blood examination reveals the following: at complete blood count (CBC), hemoglobin (Hb) 11.9 g/dL, mean corpuscular volume (MCV) 88 fL, normal platelets and leukocytes including the differential count; good glycemic control, stable renal function; values of both C-reactive protein (CRP) and serum ferritin are reported in the normal range (0.5 mg/dL and 43 μg/L, respectively). The following questions arise: 1) Is this patient actually anemic? 2) If yes, is this (mild) anemia clinically relevant? 3) Is this anemia correctable? and 4) Will correction of anemia improve the overall prognosis?

## Background: the demographic drive

The world's population is rapidly aging because of the combined effects of increase in life expectancy and falling fertility rates. The World Health Organization (WHO) estimates that the number of people aged >60 years will rise from 900 million in 2015 to 2 billion in 2050, moving from 12% to 22% of the global population (http://www.who.int/mediacentre/factsheets/fs404/en/). Noteworthy, the so-called “oldest old,” commonly defined as those aged >85 years, represent the fastest-growing segment of western populations,^1^ which in turn is the most susceptible to disease and disability. Decline of Hb levels has long been considered an almost inevitable consequence of aging, so that the term “anemia *of* elderly” has been largely accepted in the past. However, in the last decades evidence has been accumulated that anemia does reflect poor health status and increased vulnerability to adverse outcomes in elderly. The term “anemia *in* the elderly” (from here designed as “AE”) is now preferable, and implicates that, at least in principle, the cause of low Hb in an elderly subject should be determined and, if possible, treated. The first step to face this problem is an adequate definition.

## Definition of AE: a still unresolved issue

While CBC is likely the laboratory test most prescribed worldwide, the Hb thresholds for defining anemia are still matter of controversy. Classically, the normal values of any parameter should consist of the 95% reference range obtained by analyzing representative populations of well-defined healthy subjects. Up to now, the only universally accepted definition of anemia in adults is that proposed by the WHO (Hb <13 g/dL in men and <12 g/dL in women), which dates back to 50 years ago.^2^ This definition has been criticized for a number of reasons, including the small number of subjects in the original dataset and inadequate/outdated methodology.^3^ Moreover, the reference population did not include subjects aged >65 years, making highly questionable the extension of such criteria to the elderly. Since then, several alternative definitions have been proposed. In 2006, Beutler used 2 large datasets, that is, the Scripps-Kaiser database, which included >24,000 subjects (4982 aged ≥70 years),^4^ and the third US National Health and Nutrition Examination Survey (NHANES III), which included 7664 subjects (1566 aged ≥70 years).^5^ Both datasets included people of different ethnicities, allowing the known lower Hb values in individuals of African descent to be taken into account.^6,7^ As compared with classical WHO thresholds, slightly higher values were proposed in Caucasian men and women aged > 60 years (<13.2 and <12.2 g/dL, respectively), while the corresponding values in older individuals of African descent were 12.7 g/dL in men and 11.5 g/dL in women.^3^ However, this approach was not devoid of limitations. In particular, the 2 datasets were derived from the general population including both healthy and unhealthy people, and the selection of “healthy” subjects for extrapolating the “normal” Hb values was largely based on other laboratory parameters (eg, CRP and creatinine) rather than on an accurate clinical evaluation.

Some geriatric authorities have recently proposed an equal Hb threshold of 12 g/dL for defining anemia in both genders.^8^ This is largely based on the longstanding notion that the Hb decline with age tends to be more pronounced in males than in females,^9,10^ which has been traditionally attributed to progressive androgen deficiency.^11^ Indeed, a major drawback of population studies specifically aimed at defining the normal Hb ranges in elderly lies in the difficulty in enrolling an adequate number of truly healthy subjects, the so-called “wellderly.”^12^ Of note, studies on relatively small cohorts of the elderly without any significant chronic medical condition have shown that the Hb decline during aging tends to be minimal or not significant,^9,11^ even in nonagenarians.^13^

A different approach, relying on the concept of *optimal* rather than normal Hb values, has been derived by longitudinal studies highlighting an association between Hb levels and unfavorable outcomes (see also the “Prevalence of anemia in elderly: a public health problem” section). For example, a study enrolling 17,030 community-dwelling subjects from the Calgary Region who were followed for 3 years showed, in those aged >66 years, an inverse J-shaped relationship between Hb and all-cause mortality. The lowest risk for mortality occurred at Hb values between 13 to 15 g/dL for women and 14 to 17 g/dL for men.^14^ Similar results have been obtained by the Cardiovascular Health Study, which enrolled 5888 community-dwelling subjects aged ≥65 years who were followed for 11.2 years, with optimal Hb values being between 12.6 to 14.4 g/dL in women and 13.7 to 15.6 in men.^15^ However, it should be recognized that the association between anemia and mortality, even independent from concurrent diseases, does not prove a causal link, because of inherent limitations of observational epidemiology.^16^ Of note, using the above-mentioned “optimal” values for defining AE would further increase the fraction of the elderly with anemia (see the “Prevalence of anemia in elderly: a public health problem” section), particularly in the oldest old subgroup, possibly leading to excessive or unnecessary diagnostic procedures.

Table 1 summarizes the pros and cons of defining AE using the different thresholds proposed. At present, none of them can be considered ideal. The WHO is launching a novel ambitious program that should revise the Hb thresholds for defining anemia in different populations, including the elderly (http://www.who.int/nutrition/events/2017-meeting-haemoglobin-concentrations-anaemia-29novto1dec/en/). For the moment the authors and others^17,18^ believe that the original WHO thresholds still represent an acceptable compromise, as we await more precise definitions in the future.

**Table 1:**
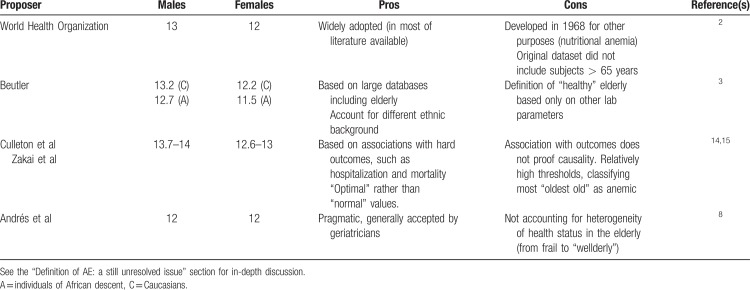
Different Hemoglobin Thresholds (g/dL) Proposed for Defining Anemia in Elderly (Pros and Cons)

## Prevalence of anemia in elderly: a public health problem

The prevalence of AE has been extensively investigated by a number of epidemiological studies in different settings.^10,14,19–24^ A systematic review of 34 studies using the WHO criteria, which included a total of 85,409 participants, has documented the following weighted mean prevalence: 12% (3–25%) in community-living, 47% (31–50%) in nursing-home residents, and 40% (40–72%) in hospitalized elderly.^25^ Analyses restricted to subjects aged >80 years found that the prevalence increased to over 25% in the community-living.^25^ Based on these numbers, it has been estimated that nearly 15 million elderly in the European Union may be anemic, and the European Hematology Association has suggested AE as a major research topic in a recent consensus document.^26^

## Clinical relevance of anemia in elderly: not an innocent bystander

**Figure 1:**
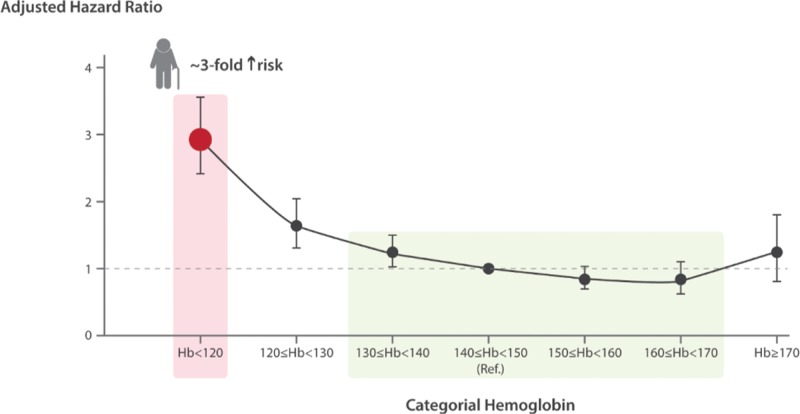
**Curve of mortality risk per Hb categories in men aged > 66 years from the Calgary Community.** The curve is J-shaped, with optimal Hb levels minimizing the risk ranging from 140 to 170 g/L (green area). Red point and arrow indicate how the patient described in the case vignette is positioned in the curve, with an ensuing mortality risk increased by 3-fold. ∗Adjusted for age, diabetes mellitus, glomerular filtration rate, and comorbidity status calculated using a validated chronic disease score. See also discussion on the “Definition of AE: a still unresolved issue,” “Prevalence of anemia in elderly: a public health problem,” and “Clinical relevance of anemia in elderly: not an innocent bystander” sections. Adapted from Culleton et al.^14^ In the original publication, the fully adjusted hazard ratio for mortality in all subjects (both men and women) older than 80 years with Hb < 110 g/L and normal glomerular filtration rate was 3.34, 95% confidence interval 2.47 to 4.51. Hb = hemoglobin.

According to epidemiological studies, AE is mostly mild, that is, with Hb around 11 to 12 g/dL. This commonly generates a misleading perception of AE as a minor problem, particularly in the setting of multimorbidity.^27^ However, AE has been associated with an impressive number of adverse outcomes, including frailty and decreased physical performance,^28–30^ reduced muscular strength with increased risk of falls,^31–34^ cognitive decline and dementia,^35–37^ increased risk of hospitalization and longer hospital stay,^14,38–40^ and even with an increased mortality risk in longitudinal studies.^14,15,39–43^ Of note, most of these associations remained statistically significant after controlling for multiple possible confounders, suggesting a negative impact of anemia per se, independently of concurrent chronic morbidities like CKD, CHF, and inflammatory disorders. Moreover, the increased risk of mortality was not confined to subjects with the most severe Hb decline, but was evident also for mild AE. For example, in men enrolled in the Calgary Study the increased risk became evident in those with Hb slightly lower than 13 g/dL, and increased sharply at Hb level of 12 g/dL^14^ (Fig. 1). As mentioned above, independent associations in observational epidemiological studies do not prove causality,^16^ and a clear direct pathophysiological link between AE and functional decline and/or mortality is still lacking. A possible mechanism might be chronic suboptimal oxygen delivery to aged and possibly already damaged organs, including the heart. Anyway, even under the most conservative hypothesis, AE would represent a robust and easily obtainable marker of serious adverse outcome, and hence has to be always seriously taken into account.

## Etiology of anemia in elderly: “Unexplained” or inadequately studied?

According to current literature based on large epidemiological surveys,^19–21,42,44,45^ AE etiology can be equally divided into 3 broad categories: 1) nutritional deficiencies, mainly represented by iron deficiency (ID), but sometimes also by folic acid and vitamin B12 deficiencies; 2) anemia of inflammation (AI), a heterogeneous group including CKD, inflammatory or infectious diseases, and tumors, extensively reviewed elsewhere,^46–48^ in which anemia is largely driven by hepcidin-induced iron sequestration into macrophages^49,50^ and cytokine-dependent bone marrow (BM) suppression; and 3) “unexplained” cases. While useful to outline the problem, this categorization has several drawbacks limiting its usefulness in clinical practice. Indeed, the above-mentioned epidemiological studies^19–21,42,44,45^ defined AE etiology merely according to few laboratory parameters, sometimes using questionable cut-off values (eg, see the “Treatment options for AE: more research is needed” section). In this way, “unexplained” AE (UAE) was essentially a diagnosis of exclusion in subjects with apparently normal values of iron/folic acid/vitamin B12 parameters, CRP, and creatinine, who lacked a more in-depth clinical evaluation. Moreover, this approach overlooks the fact that dissecting the etiology can be much more difficult in the elderly than in younger subjects, as AE is often multifactorial due to multiple concomitant morbidities. In fact, multimorbidity increases substantially with age, so that, for example, nearly 40% of patients aged >80 years have ≥4 concomitant diseases,^27^ which frequently include conditions in themselves associated with anemia, such as CKD^51^ or CHF.^52,53^ Classical diagnostic algorithms for anemia such as those based on MCV^54^ are designed to identify single etiology, but have limited value in multifactorial AE.^55^ In this scenario, adequately pointing out a potentially treatable cofactor, that is, any micronutrient deficiency, can be especially challenging.

UAE is a heterogeneous category reflecting our inadequate approach to the problem and includes several different conditions, which are not necessarily mutually exclusive in an individual patient. One possibility is that a fraction of UAE is related to the initial phase of certain myelodysplastic syndromes (MDS), that is, a group of clonal hematopoietic disorders that most commonly occur in the elderly, with a median age at diagnosis in most series of ≥65 years.^56^ Indeed, isolated anemia is frequently the first clinical manifestation of low-risk MDS,^57^ but many suspected cases do not undergo invasive definitive testing (ie, BM morphology examination and related molecular studies), especially when anemia is mild and relevant comorbidities occur.^58,59^ In such cases, a watchful waiting approach is generally preferred. Epidemiological studies on AE underestimate MDS by design,^59^ so that MDS have been claimed to account for a substantial fraction of UAE cases. However, small studies in which UAE patients were appropriately investigated concluded that MDS were present in <10% to 15% of cases.^44,60,61^ In the future, the increasing application of noninvasive testing such as next-generation sequencing (NGS) analyses of peripheral blood cells,^62–65^ as well as refined diagnostic criteria,^66^ could help in estimating the true prevalence of low-risk MDS among UAE patients. This may be particularly important considering that anemia in low-risk MDS can be sometimes corrected using erythropoietin (EPO)^67^ or innovative agents that interfere with transforming growth factor beta superfamily inhibitors of erythropoiesis, such as the activin receptor IIA ligand trap luspatercept.^67,68^

Other conditions that may underlie UAE are summarized in Table 2, and briefly discussed in the paragraphs below.

**Table 2:**
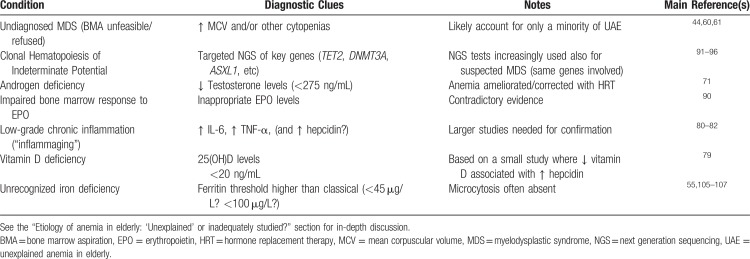
Conditions That May Underlie “Unexplained” Anemia in Elderly

### Androgen deficiency

Androgen deficiency is a plausible cofactor of UAE,^69^ especially in senior men.^70^ A recent randomized placebo-controlled trial including older men with low testosterone levels (<275 ng/dL) and mild UAE (Hb > 10 g/dL) found that administration of testosterone gel (1%) for 12 months was more effective in correcting anemia than placebo (in 58% vs 22% of cases, respectively; *P* = 0.002).^71^ Interestingly, androgen therapy also ameliorated anemia due to ID, likely because of the known ability of testosterone to suppress hepcidin production^72,73^ and thus subsequently increase iron absorption and mobilization from stores to erythropoiesis. The relatively small sample size of each anemia subgroup implies the need for confirmation by larger studies.

### Vitamin D deficiency

The elderly are at high risk for vitamin D deficiency,^74^ particularly when multimorbidity influences lifestyle and substantially reduces outdoor activities.^75^ In a large study of community-dwelling individuals, low vitamin D levels were strongly associated with anemia.^76^ Several mechanisms have been proposed, including modulation of proinflammatory cytokines,^77^ blunted response to EPO,^78^ and modulation of hepcidin levels,^79^ but further studies are needed before proposing vitamin D repletion as a possible strategy to improve AE.

### Inflammaging

This term designates a chronic low-grade inflammatory state thought to be driven by the accumulation of endogenous altered or damaged molecules with aging, and by the increased formation of reactive oxygen species.^80^ This chronic up-regulation of pro-inflammatory cytokines appears to be related to activation of the NF-κB/inflammasome pathway,^81^ and has been linked to immunosenescence and other age-associated conditions like sarcopenia, cognitive decline, and frailty,^81,82^ which in turn are associated with AE.^83^ In this case, anemia could be, at least partly, driven by a cytokine-induced increase in hepcidin, leading to iron-restricted erythropoiesis because of iron sequestration into macrophages.^46,50,84^ While hepcidin levels do not seem to increase in the general elderly population,^85^ 2 small studies in selected patients with UAE demonstrated the presence of increased hepcidin levels.^44,86^ This would corroborate this attractive hypothesis, but larger studies are needed to clarify this area.

### Altered EPO homeostasis

A progressive resistance of aging erythroid precursors to EPO^87,88^ has been proposed as a plausible mechanism for UAE. This likewise has been attributed to the known effects of many pro-inflammatory cytokines, which can also directly decrease BM responsiveness.^46,47^ Others have suggested an insufficient production of EPO by the aging kidney.^89^ However, data are inconclusive,^90^ and, in particular, EPO treatment cannot be at present recommended for UAE.

### Age-related clonal hematopoiesis

Emerging evidence indicates that changes in the hematopoietic system with aging (declining blood cell output, alterations in chemokine/cytokine levels and in the BM microenvironment) are largely due to selection of mutant clones of hematopoietic stem cells (HSC).^91^ Age-related clonal hematopoiesis can be detected by NGS studies of peripheral leukocytes, showing the presence of somatic mutations in certain key-genes (ie, *DNMT3A*, *TET2*, *ASXL1*, and others) also involved in hematologic malignancies.^91,92^ Such mutations are present in nearly 10% of otherwise healthy subjects aged 70 years (a condition named “clonal hematopoiesis of indeterminate potential” [CHIP]), and their prevalence tends to increase with further aging.^93,94^ They give rise to clones with competitive proliferating advantage over normal HSC which result in less effective erythropoiesis, possibly leading to anemia.^93^ Preliminary molecular studies in elderly with unexplained cytopenias support the hypothesis of clonal hematopoiesis as the underlying phenomenon in a fraction of UAE.^95^ Subjects with anemia and a single clonal mutation (a condition named “clonal cytopenia of undetermined significance” [CCUS]) do not fulfill all the criteria for a diagnosis of MDS,^96^ although the acquisition of further mutations confers a risk of developing hematologic malignancies.^91,93^ Noteworthy, subjects with CHIP have an increased mortality risk that is mainly driven by cardiovascular events rather than by hematological malignancies.^94,97^ Elegant studies in mice have demonstrated that *TET2*-deficient macrophages are more inflammatory than macrophages that are not derived from a mutant clone, and can accelerate atherosclerosis.^97–99^ Theoretically, a CHIP-driven systemic pro-inflammatory state could also contribute independently to the “inflammaging,” and the possible relationship between these 2 conditions represents(?) a stimulating field for future research. A possible caveat is that measurement of cytokine levels in the peripheral blood does not always accurately reflect subclinical pro-inflammatory conditions, particularly at the local BM level.^90^

### Unrecognized iron deficiency

ID represents the most frequent single cause of anemia worldwide, and a global health problem.^100^ Elderly with multimorbidity are at high risk of ID because of malnutrition, reduced iron absorption (possibly aggravated by frequent use of proton pump inhibitors [PPI]^101^), and gastrointestinal blood loss because of increased incidence of angiodysplasia and tumors, often aggravated by concomitant antithrombotic therapies.^55^ ID in elderly is often multifactorial and overlooked, since the diagnostic thresholds of traditional laboratory parameters do not perform as well as in younger patients.^55^ For example, microcytosis is not a reliable marker of ID in elderly. In a series of 4117 anemic patients aged ≥65 years, only 26.9% of those with absolute ID had a reduced MCV, while MCV was normal in 68.9%, and increased in 4.2%.^21^ Others have reported similar results, with microcytosis being present in <30% of elderly with documented iron deficiency anemia (IDA).^44,102^ The same reasoning applies to ferritin, the most reliable marker of ID, which is difficult to interpret in the elderly since levels tend to increase with age itself,^103^ not only because of inflammatory comorbidities. Thus, it is important to note that classical ferritin thresholds for IDA in younger (≤15–20 μg/L) cannot be automatically used in elderly. The classical epidemiological studies mentioned above^19,20,86^ used such cut-offs to estimate with certainty IDA as the cause of nearly 30% of AE. However, besides low ferritin, 2 other major criteria are commonly accepted as diagnostic for absolute ID: absence of stainable iron in the BM; and correction of anemia upon iron administration.^104^ The first is still considered the “gold standard” for ID, although is rarely used because of invasiveness. Regarding this criterion, a small but remarkable study in which a cohort of elderly anemic subjects underwent systematic BM aspiration found that the probability of IDA was maximal (96%) when ferritin was <18 μg/L, but still very high (64%) for ferritin values between 18 and 45 μg/L.^105^ Similar results have been obtained in other small studies where the diagnostic yield of ferritin versus absence of BM stainable iron was evaluated. Punnonen and colleagues reported an optimal diagnostic efficiency for a ferritin threshold of 41 μg/L.^106^ Karlsson reported an even higher ferritin cut-off in the elderly, that is, 87 μg/L.^107^ Overall, these studies strongly suggest that ferritin thresholds for IDA in people aged >65 years should be reconsidered. In our opinion, it could be reasonable to consider a threshold of at least 45 μg/L, if not 100 μg/L,^55^ particularly when certain comorbidities occur, such as stage 3 to 5 CKD^108–110^ or CHF.^111–113^ Regarding the second diagnostic criterion mentioned above (anemia correction with iron supplementation^104^), an important lesson has recently come from studies on patients with CHF, in whom a mild anemia is present in up to 50%.^52,53,112,114^ Anemia in CHF recapitulates some features of AE since the pathogenesis is typically multifactorial due to concurrence of ID,^111^ low-grade subclinical inflammation,^115^ decreased renal function, and hemodilution.^114^ Seminal studies on iron supplementation in CHF patients, most of them being elderly, used a broad definition of ID, that is, ferritin levels <100 μg/L, or up to 300 μg/L if concomitant transferrin saturation was <20%.^116,117^ While the main goal was to correct tissue ID (cardiac and muscular, not necessarily the anemia), a subanalysis on patients who were anemic at baseline showed significant amelioration of anemia after intravenous (i.v.) iron.^118^ This corroborates the validity of the unusually broad ferritin thresholds used to define ID, notwithstanding their substantial difference from those classically considered. Such results have been consistently replicated,^119,120^ so that most recent and authoritative guidelines agree on suggesting i.v. iron treatment of CHF patients when ferritin is <100 μg/L.^121–123^ Altogether, these data on BM iron stores and anemia correction with iron highlight the need of using ad hoc ferritin thresholds in AE that are consistently higher than those used in younger people. To establish precise thresholds would require extensive studies.

**Figure 2:**
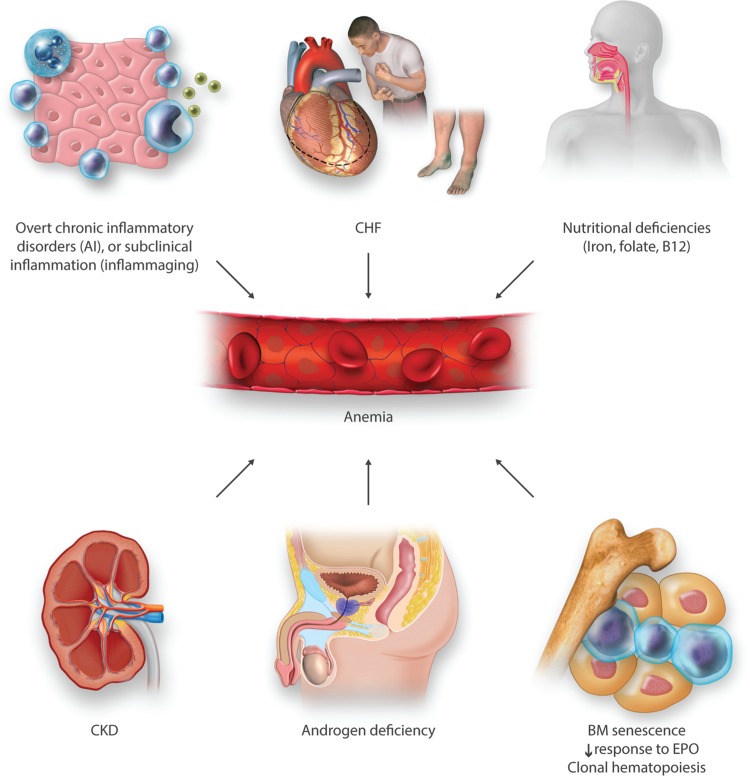
**The complex and multifactorial pathophysiology of AE.** Some of the major factors contributing to AE etiology are illustrated. More than 1 condition is frequently present in individual patients, particularly in those with multimorbidity. See also discussion on the “Etiology of anemia in elderly: ‘Unexplained’ or inadequately studied?” section. AE = anemia in elderly.

Figure 2 summarizes the main conditions involved in the complex multifactorial pathogenesis of AE.

## Treatment options for AE: more research is needed

At present, a reliable treatment can be proposed only for a fraction of AE patients, particularly those with nutritional AE through correction of hematinic deficiencies (most frequently iron, but sometimes also folic acid and vitamin B12). This appears particularly important as longitudinal studies suggest that such patients are actually those at the highest risk of subsequent mortality.^42^ When macrocytosis (MCV >100 fL) is present, folic acid and vitamin B12 deficiencies should be investigated. Serum levels of folate and vitamin B12 may be clearly normal (ie, >4 ng/mL or >300 pg/mL, respectively), borderline (2–4 ng/mL or 200 to 300 pg/mL, respectively), or definitively low (<2 ng/mL or <200 pg/mL, respectively). Borderline results require additional testing (measurement of methylmalonic acid and homocysteine) to determine the accuracy of the diagnosis, and, if confirmed, the cause (ie, autoantibodies to intrinsic factor and/or to parietal cells) (for extensive reviews see Antony^124^ and Stabler^125^). Of note, the median age range of pernicious anemia (the most common cause of severe vitamin B12 deficiency, associated with achlorhydria) is 70 to 80 years, and milder form of atrophic gastritis with hypochlorhydria can affect up to 20% of older adults.^125–127^ While intramuscular vitamin B12 monthly injections are usually recommended for vitamin B12 deficiency, high dose (1000–2000 μg/day) oral cobalamin tablets are increasingly popular and equally effective.^125,128^

Regarding IDA, the new i.v. iron preparations appear particularly attractive in the elderly, because of the easy schedule (infusion of the total therapeutic dose in a single injection) and the reassuring safety profile.^100,113^ Ferric carboxymaltose (FCM) has been proven particularly effective in ID anemic elderly with CHF.^116–118^ By contrast, in a recent trial, oral iron was ineffective,^129^ which was attributed to increased hepcidin levels occurring in the pro-inflammatory state that characterizes CHF.^115^ Results in the CHF setting may have more general implications for treating ID in the elderly, for several reasons^55^: 1) compliance with oral iron, which often requires at least 3 months administration, can be difficult in the setting of polypharmacy^130,131^; 2) elderly patients may have reduced iron absorption, because of increased frequency of hypochlorhydria (see above), frequent PPI prescription, and/or increased hepcidin levels driven by concomitant inflammatory disorders or CKD^132,133^; and 3) single-dose infusions avoid multiple hospital visits, which could be particularly cumbersome in elderly with reduced mobility. In the future, measuring hepcidin levels could facilitate the selection of the more appropriate route of iron administration in ID elderly.^72,134^ A possible algorithm anticipating this scenario has been proposed elsewhere.^55^ Nevertheless, ad hoc clinical trials are needed to confirm the safety and efficacy of a more widespread use of i.v. iron formulations in the elderly. Similarly, it will be particularly important to know, through longitudinal observations, whether or not anemia correction improves hard outcomes like risk of hospitalization and mortality, thus confirming its causal role.

In AE patients without hematinic deficiency, treatment is more difficult, but some options may emerge in the near future. In AI, treatment should mainly be aimed at controlling the underlying condition(s). Nevertheless, a number of hepcidin inhibitors are being developed (for extensive reviews see Poli et al^135^ and Ruchala and Nemeth^136^), including spiegelmer lexapeptide NOX-H94,^137,138^ anticalin PRS-080,^139,140^ heparin derivatives,^141–143^ and other agents interfering with Bone Morphogenetic Protein 6 hepcidin activating pathway as CSJ137 (https://clinicaltrials.gov/ct2/show/NCT02570854).

A single study has reported beneficial effects of Epoetin alpha in African American women with UAE.^144^ Theoretically, by analogy with certain low-risk MDS,^145^ EPO treatment could be proposed for AE in the setting of CCUS and documented low EPO levels. However, the use of EPO analogs in AE remains off-label, and there are safety concerns regarding the risk of thromboembolic complications. In particular, their possible use should not be aimed at correcting AE, but rather to maintain a Hb threshold of 11.5 g/dL.^109^ Novel agents able to increase endogenous EPO levels by Hypoxia-Inducible Factor Prolyl Hydroxylase Inhibitors,^146^ now in study for anemia in CKD, might represent a future option in selected patients.

As mentioned above, androgens may have a role in AE, in men with documented low testosterone levels.^71^ The overall health benefits of a more widespread hormone replacement therapy in older men remain to be established.

Blood transfusions remain the only feasible option for elderly with severe, symptomatic anemia. Aside from this scenario, use of transfusions should be kept at minimum because of inherent risks.^147^ A comprehensive review including recommendations about transfusions in elderly has been recently reported elsewhere.^148^

## Going back to the case vignette: how the patient could be classified and how we would manage him

As regard to the question no. 1 (Is this patient actually anemic?), this patient can be actually considered anemic, regardless of the definition of AE we would apply (see Table 1 and the “Definition of AE: a still unresolved issue” section). Question no. 2 (Is this mild anemia clinically relevant?): his anemia, even if mild (Hb 11.9 g/dL), has to be considered clinically relevant, because of the increased risk of further hospitalizations and also death. This risk appears independent of the numerous comorbidities, and can be estimated near 3-fold as compared to that of a similar patient with Hb values within the optimal range (Fig. 1). Question no. 3 (Is this anemia correctable?): this patient has likely an absolute ID, even if the serum ferritin level is reported as “normal” by the reference laboratory (see the “Etiology of anemia in elderly: ‘Unexplained’ or inadequately studied?” section). Because this patient is concomitantly affected by CHF with reduced ejection fraction, we would definitively treat him with a single infusion of FCM (1 g), according to current guidelines issued by the European Society of Cardiology, the American Heart Association, and the Canadian Cardiovascular Society.^121–123^ A delicate question arises on whether or not (and if yes, how) the cause of ID should be investigated in this patient with mild anemia and “pseudo-normal” ferritin levels. According to guidelines from the British Society of Gastroenterology,^149^ all “adult” males with IDA should undergo upper and lower gastrointestinal endoscopy, but no specific recommendation is given for elderly subjects. The only guidelines on ID that specifically mention the elderly are those issued by the British Columbia Medical Association (available online at https://www2.gov.bc.ca/gov/content/health/practitioner-professional-resources/bc-guidelines/iron-deficiency). In a short paragraph, they recommend investigation of the cause of ID in the elderly “if the life-expectancy is >1 year.”^150^ In our opinion, in a frail multimorbid elderly patient such as the one illustrated in the vignette, any decision should be made on a case-by-case basis. We would search for additional information to evaluate the risk of a gastrointestinal malignancy. If, for example, family history is negative for gastrointestinal malignancy, any previous colonoscopy performed for colon cancer screening did not provide evidence for a concerning lesion, no alarming sign (eg, weight loss or changes in evacuation habit) is present, and no mass is palpable on abdominal examination, we would probably opt for a cautious observation after discussion with the patient and the caregivers. Review of previous CBCs showing a relative stability, and a dietary history suggesting a poor iron intake would likely reinforce this choice. Question 4 (Will correction of anemia improve the overall prognosis?): in this patient, suffering from CHF among the other comorbidities, there is sufficient evidence that IDA correction could actually improve the overall prognosis.^119^ Aside from this specific setting, no similar evidence exists in other cases of AE with proven ID. Much less evidence exists for any other AE subgroups, including AI or UAE. Clinical studies should be performed to address this critical issue.

## Concluding remarks

AE represents a potential public health crisis, because of the high prevalence involving millions of persons. Moreover, even mild AE is independently associated with hard outcomes including quality of life (QoL), hospital admissions, and reduced survival. Thus, it needs to receive adequate attention in clinical practice, and not be merely considered a “physiological” consequence of aging.

The pathogenesis of AE is complex and quite often multifactorial. Dissecting the cause(s) can be difficult because of inapplicability of traditional algorithms for single-cause anemia in the young, lack of consensus on ad hoc laboratory values, confounding factors, and multimorbidity. At least one-third, and likely more, of AE cases are due to ID, which could be relatively easily corrected by iron supplementation, particularly using the new i.v. single-dose iron formulations. There is uncertainty regarding the appropriateness of searching intensively the cause of ID in the elderly, particularly in the frail “oldest old.” A personalized approach with careful balance of the risk/benefit between over-diagnosis/over-treatment versus under-diagnosis/under-treatment should be applied. Aside from the setting of concomitant CHF, rigorous and well-designed clinical trials are needed to prove the efficacy of iron not only to correct anemia, but also to improve major outcomes such as QoL, hospitalization, and survival. Similarly, further research is needed to evaluate the actual benefit of correcting AE in different settings, that is, when inflammation plays a major role, using emerging approaches like antihepcidin agents.

It is time to promote and coordinate multidisciplinary expertise on AE, to initiate high-quality research, and to facilitate networking with stakeholders and policy makers.
